# Ethical considerations for respectful research participant payment processes

**DOI:** 10.1017/cts.2024.650

**Published:** 2024-11-04

**Authors:** Devan M. Duenas, Elliott Mark Weiss, Benjamin S. Wilfond, Stephanie A. Kraft

**Affiliations:** 1 Seattle Children’s Research Institute, Treuman Katz Center for Pediatric Bioethics and Palliative Care, Seattle, WA, USA; 2 Department of Pediatrics, Division of Bioethics and Palliative Care, University of Washington School of Medicine, Seattle, WA, USA; 3 Department of Pediatrics, Division of Neonatology, University of Washington School of Medicine, Seattle, WA, USA; 4 Department of Bioethics and Decision Sciences, Geisinger College of Health Sciences, Danville, PA, USA

**Keywords:** Research ethics, respect, payment, equity, person-oriented ethics

## Abstract

**Background:**

Researchers and research organizations acknowledge the importance of paying research participants but often overlook the process of providing participant payments as a locus for improving equity and inclusion in clinical research. In this conceptual paper, we argue that participants’ lived experiences and social context should be recognized and respected when developing these processes.

**Methods:**

We consider how participant payment processes that require specific payment types, delay the timing of payment, or require sharing sensitive information may impose barriers to equitable research. Building on findings from empirical research of participants’ perspectives on respect in research and a relational ethics framework of person-oriented research ethics, we explore how researchers and research organizations can better demonstrate respect through the research participation payment process.

**Results:**

We propose five considerations for demonstrating respect when providing payment: (1) practice cultural humility, (2) be mindful of socioeconomic factors, (3) be flexible, (4) be transparent, and (5) maintain open communication. These considerations are intended to address the lack of existing ethical guidance around the process for participant payments and promote more inclusive clinical research. We provide a set of sample questions for research teams to consider how they could modify their payment processes to better demonstrate respect.

**Conclusions:**

By better demonstrating respect for participants when providing payment, researchers can work toward ensuring that their research procedures are more inclusive, respond to the needs of diverse communities, and result in more equitable relationships with participants.

## Introduction

Research organizations and research funders are increasingly implementing strategies to improve equity, diversity, and inclusion in clinical research, recognizing that recruitment and retention of patients with diverse lived experiences is an essential step toward health equity [1–3]. However, institutions may have several structurally imposed barriers that prevent equitable participation in clinical research [4–6]. To overcome these barriers, it is crucial for researchers to address those areas that are within their purview.

The process of research participant payment is one area where researchers could create more inclusive procedures that are responsive and respectful of the community’s needs and the barriers to participation in clinical research. For the purposes of our analysis, clinical research encompasses research conducted with patient populations about healthcare including social science and behavioral surveys and interviews, observational studies, and clinical trials for drugs, devices and behavioral interventions. Providing payment serves several purposes, including to support recruitment and retention, to reimburse for expenses or lost wages and thereby reduce the financial burdens of participation, and to compensate participants for their time and inconvenience [7]. Limited attention, however, has been paid to the *process* of participant payments. That is, not *whether* or *how much* participants are paid, but *how* they receive payment, e.g., what additional steps they need to take to receive payment, what forms of payment they can receive, and what logistics are required throughout the process.

Failure to implement equitable payment processes can result in the exclusion of marginalized communities. For example, requiring collection of social security numbers could result in the exclusion of undocumented immigrants or their families, using debit card vendors whose cards have penalty fees could come as a surprise and create an unnecessary barrier for low-income families, and requiring documentation of a physical address could result in the exclusion of individuals without permanent housing. Institutional policies often dictate the forms of payment that can be issued (e.g., e-gift cards, reloadable debit cards) and the participant information required to be collected to receive payment (e.g., social security number, mailing address), which can create or exacerbate these issues which disadvantage some communities and result in the exclusion of others [8–9]. Examining how these processes and policies impact participants offers a valuable opportunity for researchers and research organizations to develop more participant-oriented policies. Doing so will facilitate approaches to payment that can improve the overall participation experience, demonstrate institutional trustworthiness, and foster more inclusive research [10].

In this paper, we examine how researchers and research organizations can develop participant-oriented payment processes. We consider this process through the lens of respect for participants and communities, and we examine practical considerations for demonstrating respect through the payment process. First, we discuss how the relationship between the researcher, the participant, and the participant’s community should be considered when developing policies and processes for payment. We then propose five considerations for demonstrating respect when providing payment. These considerations are intended to address the gaps of current policies and processes and improve equity with research payments. Finally, we provide sample questions, based on relevant factors under each consideration, research teams could engage with to begin to think about how they could modify their payment processes to better demonstrate respect and identify areas in need of further empirical study.

### Payment and the principle of respect

Ethical debate about payment for research participation has largely focused on whether payments can exert undue influence, clouding a research participant’s judgment and their evaluation of the benefits and risks of a study [11–14]. These concerns for participant autonomy during the decision-making process represent efforts directed toward demonstrating respect for participants when providing payment [15]. However, this focus on autonomy does not fully account for participants’ experiences of respect during a research study, nor does it capture that for marginalized communities demonstrating respect may require overcoming barriers that have historically limited their participation. When thinking about interactions with participants, a broader definition of respect in which regards participants as distinct individuals with their own unique experiences and, in the actions taken, considers their needs, interests, feelings, and the challenges they face [16]. As Phillips argues in a critical analysis of exploitation in payment [17], it is important to acknowledge that participants may experience payment differently from how the researchers intend, and the benchmark for fair payment should be based on that experience. Otherwise, researchers risk relying on a set of assumptions about payment that may not reflect participants’ lived realities as recipients of the payment, especially for participants whose backgrounds differ from those of the researchers.

Recent efforts at understanding how best to demonstrate respect for participants in clinical research have contributed to a more expansive understanding of respect in the research setting. For example, Kraft and colleagues conducted exploratory qualitative interviews with an ethnically and socioeconomically diverse group of participants in a clinical genomics implementation study, identifying four domains for demonstrating respect: empathetic interactions, open communication, understandable materials and accessible procedures, and transparent and neutral consent processes [18]. Building on this and other literature on informed consent and respect for persons in the context of pragmatic clinical trials, Morain and colleagues proposed eight dimensions of demonstrating respect for persons to guide the ethical design, conduct, and oversight of pragmatic clinical trials [19]. These dimensions include (1) engaging patients and communities in research design and execution, (2) promoting transparency and open communication, (3) maximizing agency, (4) minimizing burdens and promoting accessibility, (5) protecting privacy and confidentiality, (6) valuing interpersonal interactions with clinicians and study team members, (7) providing compensation, and (8) maximizing social value. Although these dimensions were developed in the context of pragmatic trials, they further expand how researchers might approach demonstrating respect in different settings.

Key to respecting participants when providing payment for research participation is addressing the relational nature of research. Building relationships rooted in the values of respect and reciprocity is critical for engaging historically marginalized communities; for example, these values are integral for many Indigenous populations [20–22]. We propose that incorporating a relational ethics approach, which focuses on the nature of the interactions between the researcher and participant and situates these interactions within each individual’s social context [23–24], is essential to thoroughly understand how payment processes contribute to participants’ experiences of respect. Specifically, we look toward the “person-oriented” research ethics approach developed by Cascio and Racine [25]. Cascio and Racine’s model proposes five practical, “person-oriented” guideposts to guide research design, recruitment, data collection, and other steps in the research process: respect for holistic personhood, acknowledgement of lived world, individualization, focus on researcher-participant relationships, and empowerment in decision-making. In the section below, we apply this “person-oriented” lens to a consider the impact of the payment process on the participant, and tailor the process to better demonstrate respect for participants.

### Considerations for demonstrating respect when providing payment

Building on the prior literature on participant perspectives on respect, respect in pragmatic clinical trials, and person-oriented research ethics [18,19,25], we propose five considerations for better demonstrating respect when providing payment for research participation: (1) practice cultural humility, (2) be mindful of socioeconomic factors, (3) be flexible, (4) be transparent, and (5) maintain a communicative relationship. Each consideration is derived by identifying overlapping elements from the prior literature and identifying a practical consideration for demonstrating respect when providing payment. These considerations aim to improve participants’ experiences of respect in research, collectively work toward advancing justice by ensuring no person is excluded due to inaccessible or unusable payment processes, and minimize any risks associated with the payment process in a participant’s unique context. Table 1 indicates the ethical foundations for each consideration.

**Table 1.** Considerations for providing respectful payment

		Underlying Ethical Rationales		
Consideration	Description	Cascio and Racine 2018	Kraft et al. 2020	Morain et al. 2022
*Practice cultural humility*	Provide payments that are sensitive to the participant’s beliefs, values, and norms. If payment is not culturally appropriate, explore alternative ways of demonstrating appreciation.	Acknowledgement of lived world	Maintaining relationships through open and accessible communication Addressing unjust structures by promoting inclusivity	–
*Be mindful of socioeconomic factors*	Provide payment that is inclusive of, accessible to, and equitable to all socioeconomic groups.	Respect for holistic personhood Acknowledgement of lived world	Addressing unjust structures by promoting inclusivity	Minimizing burdens and promoting accessibility
*Be flexible*	Accommodate participants’ evolving needs by allowing for different types and timing of payments.	Acknowledgement of lived world Individualization	–	Minimizing burdens and promoting accessibility
*Be transparent*	Provide participants with clear information about the process and requirements for payment.	Empowerment in decision	Promoting autonomy through transparent and neutral consent processes	Promoting transparency and open communication Maximizing agency
*Maintain open communication*	Create open channels for communication about the process, including the timing of payments, with participants throughout the duration of the study.	Focus on researcher-participant relationships Individualization Empowerment in decision	Maintaining relationships through open and accessible communication	Promoting transparency and open communication Valuing interpersonal interactions

Individually and collectively, these considerations are intended to aid researchers and research organizations to develop payment processes that better demonstrate respect. While external barriers may limit an individual researcher or team from fully incorporating all of these considerations, we encourage individuals to strive to fulfill as many as possible and research leaders to advocate to incorporate these into policy change.

### Practice cultural humility

Researchers should approach the process of providing payment with a mindset of cultural humility. Tervalon and Murray-García summarize that “cultural humility incorporates a lifelong commitment to self-evaluation and critique, to redressing power imbalances in the physician-patient dynamic, and to developing mutually beneficial and non-paternalistic partnerships with communities on behalf of individuals and defined populations” [26]. Similarly, a conceptual analysis by Foronda and colleagues defines cultural humility as a “process of openness, self-awareness, being egoless, and incorporating self-reflection and critique after willingly interacting with diverse individuals” with the results being mutual empowerment, respect, partnerships, optimal care, and lifelong learning [27].

Approaching the payment process with cultural humility can help researchers navigate cultural norms and find ways to provide payment to participants that is respectful of their values and traditions. For instance, researchers should select and procure payments that are culturally appropriate in recognition of participants’ beliefs, values, and norms. While it may be acceptable in many cultures to receive some form of payment for participating in research, the specific type of payment deemed acceptable may vary from culture to culture. For example, a gift card to a steakhouse may not be appropriate for a community with religious or other dietary restrictions against consuming meat. If receiving payment for research participation is not viewed as acceptable, researchers could explore alternative ways of demonstrating appreciation for the participant’s or the community’s contributions to the study. Some alternatives may be to provide a thank you card, acknowledgements in articles and presentations, or inviting participants to share their experience at conferences or other study-related events, all of which could be offered to participants regardless of whether their community finds payment acceptable. Ongoing engagement with participants could also be helpful for establishing culturally acceptable forms of conveying gratitude for future research endeavors.

Openly communicating what information needs to be collected from participants to receive payment is also important, as some communities may be reticent to share certain types of information due to potential exposure to risk or discomfort with sharing sensitive information. Here researchers should engage with communities to share the reasons for mandatory collection and to learn how best to collect necessary information in a way that respects their concerns, for example, prioritizing privacy for individual conversations about payment. Researchers may want to work with their institutions or funders to make sure that potentially sensitive information is collected only where necessary.

### Be mindful of socioeconomic factors

Researchers should identify and use payment types that are inclusive and equitable across all socioeconomic strata. Given that some types of payment have specific requirements of participants (e.g., collecting sensitive information, accessing technology, accessing financial institutions), researchers should be mindful that using these types of payment could lead to the exclusion of potential participants, or even entire segments of the population. Payments should be provided to participants in a form that is not only useable but also useful as defined by participants. Payments should also be provided in a way that reduces participant burden by eliminating processes that are overly complicated, inconvenient, or perpetuate injustice against marginalized communities, and researchers should consider how the payment process may, or may not, fit with participants’ daily lives and activities. For example, gift cards to a store without a local presence may lack value, and reloadable gift cards that require bank accounts to withdraw the funds in cash would demonstrate a lack of consideration for those unbanked. Additionally, determining how best to provide a participant with their payment so that it is received in a timely and efficient manner is important, as it may help offset burdens incurred as part of the research.

### Be flexible

Researchers should consider how they can be flexible with the timing or types of payment provided to participants. To the extent feasible, researchers should provide payment at a time that is most convenient for the participant. For example, research teams should be prepared to hand participants’ their payment at the end of a study visit. Alternatively, if the participant prefers not to be handed payment at the end of a study visit, teams could provide the option to have the payment sent at a later time.

Flexibility with the type of payment provided should also be considered when determining how to best demonstrate respect. Researchers should consider not only providing options for the types of payment a participant could receive but could allow participants to choose different types of payments. For studies using gift cards, whether physical or electronic, researchers should be prepared to address issues with mailing gift cards or a participant’s inability to access e-gift cards by knowing all the options available to them and working with their institution to have these ready. This degree of flexibility demonstrates the team’s understanding that a participant’s situational context may change (e.g., loss of employment, experiencing homelessness) and the team’s willingness to accommodate these unforeseeable circumstances. We acknowledge that teams must work within the parameters of their institution’s policies, and that being flexible for participants will require balancing factors such as feasibility and cost.

### Be transparent

Researchers should strive to be as transparent as possible about payment and the process of providing it to participants, maintaining their efforts to approach communication from a point of cultural humility. This transparency could start as early as with recruitment materials that indicate the amount being provided and types of payment being offered. Researchers should maintain transparency throughout the study with the next step being through the informed consent process. According to the Food and Drug Administration’s Office of Good Clinical Practice, “All information concerning payment, including the amount and schedule of payment(s), should be set forth in the informed consent document” [28]. The consent process should also disclose any requirements for receiving payment such as the mandatory collection of personal data (e.g., physical mailing address, date of birth, social security number) and any relevant information about potential tax implications. Throughout the study, researchers should continue to make participants aware of any institutional policies regarding payment as certain policies or procedures could negatively impact a participant’s ability to receive or use the payment offered through the study. When developing communication materials researchers should bear in mind the health literacy of the communities they are engaging, using plain language and developing visual aids where appropriate. When speaking with participants, researchers should also provide a safe and private environment with which to hold a conversation and engage participants with empathy and open-mindedness.

### Maintain open communication

Researchers should maintain communication about the process, including the timing of payments, with participants not only during the consent process, but throughout the course of the study. Establishing and maintaining an open and accessible line of communication with participants allows the study team to discover and understand the participant’s needs and concerns about payment. To ensure that participants can communicate these needs and concerns, researchers should provide multiple avenues for participants to reach the study team. Having at least a phone number and an email address, but ideally other methods of contact (e.g., a mailing address, texting, and social media), would allow for the greatest range of accessibility for participants especially for those who may be more comfortable with specific forms communication. Researchers should also provide participants with prompt reminders and follow-ups regarding the issuance of their payment (e.g., incentives, participation-related compensation) and should keep participants apprised of any updates or delays.

Researchers should be mindful of the relationship that is being built between themselves and the participants and treat the relationship as more than a transaction. In this way, researchers should consider providing individualized support for participants should they require assistance with payments. Study team members who actively engage with participants should have access to all study information regarding payment so that they are able to communicate that effectively with participants.

Researchers should also consider taking more proactive approaches to addressing any issues with payment that may arise. Rather than relying on participants to identify issues and bring them to the attention of the research team, researchers should maintain accountability for ensuring that the payment process is effective and efficient for participants and actively monitor their payment processes to identify and address issues before they impact the participant. Similarly, when unforeseen issues do arise, research teams should not just be reactive but responsive; that is, they should apologize when appropriate, work with the participant to resolve the issue rather than tackling the problem without engaging the participant, and make the necessary improvements to the process to avoid future issues.

### Consider these issues before starting recruitment

To successfully implement these considerations, we suggest addressing each of these five considerations prior to beginning recruitment. If researchers have experience in their methods and working with their target participant population, this may be a relatively straightforward process. In some cases, a more active community engagement process should be considered. This process may take different forms depending on the community. In any case, however, engaging as early possible is likely to be most beneficial for the research team and the community. Community engagement often involves the research team working closely with a diverse group of individuals from the community or communities from which they plan to recruit to discuss the study, soliciting feedback about (or co-creating) the study’s procedures and policies, and collaborating with community members to address concerns [29–30]. This could take multiple forms, for example, working with patient advocacy committees, community advisory boards, and participant representatives at the study design stage, and/or building on prior experiences with the communit [31]. Community engagement about payment should encompass discussions about the role of payment, as well as partnerships with communities to understand payment’s impact on its members, and joint decision making about the types of payment that will be offered, the processes for providing these payments, and how this this information will be conveyed to participants. Tailoring the research approach to fit the community’s needs is an important way that researchers can demonstrate respect for communities. In conjunction with the considerations proposed here, ongoing community engagement should also be considered for monitoring and tailoring payment processes as a study progresses, in addition to other study considerations.

### Addressing potential concerns with our approach

Our approach is premised on the ethical importance of demonstrating respect in the process of payment, which itself depends on the ethical consensus that payment for research participation is appropriate in at least some cases. Some may have concerns that adequate payments to research participants may undermine participants’ decision-making. Although the question of amount or fact of payment is outside the scope of this paper, our considerations are intended to ensure that no participant experiences a lack of respect in a study due to limits on how they can use a payment. That is, once payment has been determined to be acceptable, the process for delivering payments must account for participants’ individual lived experiences.

Another set of potential concerns may be that integrating these considerations could add administrative burden and additional costs that would negatively impact studies. While these efforts will take time and resources, we assert the ethical obligation of researchers to demonstrate respect for participants and their communities can justify these efforts. Further, these efforts may be beneficial for establishing, or reestablishing, trust with communities, especially those that have been historically marginalized or excluded from research. Building respectful and trusting relationships with communities is critical for conducting more inclusive and equitable research.

These considerations can demonstrate to participants that the research team is attentive to cultural norms and practices, someone’s financial struggles or that someone’s life circumstances could change, or to need for open and honest communication. However, we also acknowledge that these considerations have limitations including any policy restrictions imposed by their institution as well as practical limitations such as not having every type of payment (e.g., cash, check, physical gift card, and e-gift card) on hand and available at all times. Research teams will need to evaluate the strengths and limitations of the considerations as they relate to their study population and apply them accordingly based on what they and participant populations find best demonstrates respect for participants.

### Applying the considerations

To aid research teams in applying these considerations, we offer questions for teams to consider when developing, implementing, and refining their payment processes. Table 2 describes each consideration, relevant factors for research teams to think about as they pertain to the study and study population, and finally sample questions for the research team to address as they relate to the payment process. This aid is not intended to be exhaustive and should be modified by the research team to appropriately address issues faced by their study population.

**Table 2.** Applying the considerations

Consideration	Relevant factors	Corresponding sample questions
*Practice ultural humility*	Cultural norms	What are the relevant cultural norms or practices regarding the receipt of payment? What factors should be considered to determine the appropriateness of a type of payment based on the culture’s values or traditions?
	Privacy concerns	Does the topic of research raise potentially sensitive issues that may heighten privacy concerns? How could the payment process be modified to alleviate these concerns?
*Be mindful of socioeconomic factors*	Financial constraints	What unique financial constraints are participants facing that could impact the receipt of payment, e.g., based on employment/student status, welfare eligibility, or other local conditions? What alterations to the payment process could be made to accommodate these constraints?
	Specific needs	What are the participants’ needs that should be factored in when developing the payment process? What modifications to the payment process could be made to reduce participants’ burden?
*Be flexible*	Timing	Can the payment schedule be adjusted to accommodate participants’ needs?
	Type of payment	Which types of payment best align with participants’ needs, e.g., related to privacy considerations or where payment can be used? Could multiple types of payment be offered as options to allow participants to choose?
*Be transparent*	Participant-facing materials	What details about payment are included in the consent form or other patient-facing materials, e.g., recruitment flyers and website? Have participants been given enough information about timing, type of payment, and information required to set appropriate expectations?
	Ongoing contact	What steps have been established to inform and update participants about payment as the study progresses?
*Maintain open communication*	Payment status	How often does the team communicate the progress of payment to your participants? How can the team be proactive in their communication with participants about payment?
	Third-party solutions	What processes has the team established to communicate with participants about payment when issuing payment through a third-party solution? How can the team most efficiently act as an intermediary between the participant and third-party solution?

## Conclusions

As researchers and research organizations strive toward greater equity, diversity, and inclusion in their research studies, it is important to consider all the factors that could impact a participant’s experience of respect including those historically overlooked processes such as providing payment. The considerations presented here reflect a preliminary examination of how to better demonstrate respect in the process of providing payment for research participation. We have proposed an aid to guide researchers in operationalizing these considerations and to prompt them to make thoughtful choices about payment methods. Moving forward, researchers should seek robust and diverse community input to revise this aid and co-create additional tools as needed. Implementing and evaluating the impact of these considerations on participant diversity and the experience of inclusion will be another important next step. Further, continued attention to participant experiences will help institutions identify—and work to dismantle—barriers for their participant communities. In doing so, they can take an important step toward more respectful and equitable research processes.
